# Importance of the Two Dissimilatory (Nar) Nitrate Reductases in the Growth and Nitrate Reduction of the Methylotrophic Marine Bacterium *Methylophaga nitratireducenticrescens* JAM1

**DOI:** 10.3389/fmicb.2015.01475

**Published:** 2015-12-24

**Authors:** Florian Mauffrey, Christine Martineau, Richard Villemur

**Affiliations:** Institut National de la Recherche Scientifique-Institut Armand-Frappier, LavalQC, Canada

**Keywords:** *Methylophaga*, denitrification, nitrate reductase, knockout, RT-qPCR

## Abstract

*Methylophaga nitratireducenticrescens* JAM1 is the only reported *Methylophaga* species capable of growing under anaerobic conditions with nitrate as electron acceptor. Its genome encodes a truncated denitrification pathway, which includes two nitrate reductases, Nar1 and Nar2; two nitric oxide reductases, Nor1 and Nor2; and one nitrous oxide reductase, Nos; but no nitrite reductase (NirK or NirS). The transcriptome of strain JAM1 cultivated with nitrate and methanol under anaerobic conditions showed the genes for these enzymes were all expressed. We investigated the importance of Nar1 and Nar2 by knocking out *narG1, narG2* or both genes. Measurement of the specific growth rate and the specific nitrate reduction rate of the knockout mutants JAM1Δ*narG1* (Nar1) and JAM1Δ*narG2* (Nar2) clearly demonstrated that both Nar systems contributed to the growth of strain JAM1 under anaerobic conditions, but at different levels. The JAM1Δ*narG1* mutant exhibited an important decrease in the nitrate reduction rate that consequently impaired its growth under anaerobic conditions. In JAM1Δ*narG2*, the mutation induced a 20-h lag period before nitrate reduction occurred at specific rate similar to that of strain JAM1. The disruption of *narG1* did not affect the expression of *narG2.* However, the expression of the Nar1 system was highly downregulated in the presence of oxygen with the JAM1Δ*narG2* mutant. These results indicated that Nar1 is the major nitrate reductase in strain JAM1 but Nar2 appears to regulate the expression of Nar1.

## Introduction

*Methylophaga* sp. are methylotrophic *Gammaproteobacteria* that are typically isolated from marine environments or brackish waters. They have a strict requirement for Na^+^ for growth and use one-carbon compounds such as methanol or methylamine (but not methane) as sole carbon and energy sources with carbon assimilation proceeding via the 2-keto-3-deoxy-6-phosphogluconate (KDPG) aldolase-variant of the ribulose monophosphate (RuMP) pathway (Boden, 2012). They all are strictly aerobic with the exception of *Methylophaga nitratireducenticrescens* JAM1, which can grow under anaerobic conditions by reducing nitrate into nitrite but does not reduce nitrite further (Auclair et al., 2010; Villeneuve et al., 2013). *M. nitratireducenticrescens* JAM1 was isolated from a denitrifying biofilm developed inside of the methanol-fed fluidized denitrification system used to treat seawater at the Montreal Biodome in Canada (Auclair et al., 2010).

The nitrate-reducing activity of *M. nitratireducenticrescens* JAM1 is correlated with the presence and expression of two distinct dissimilatory Nar nitrate reductases (Nar1 and Nar2; Auclair et al., 2010; Villeneuve et al., 2013). Nar nitrate reductases are membrane-bound enzymes in which the catalytic subunit faces the cytoplasm and catalyzes the reduction of nitrate into nitrite for energy production (Bonnefoy and Demoss, 1994). They are multimeric enzymes typically encoded by the *narGHJI* operon with *narGHI* encoding the subunits of the enzyme. (Gonzalez et al., 2006). The expression of the *narGHJI* operon is induced by a high concentration of nitrate and low concentration of oxygen. However, many counterexamples exist. In *M. nitratireducenticrescens* JAM1, the *narG1* and *narG2* genes have been shown to be expressed in the presence or absence of nitrate and oxygen in pure cultures, which indicates putative constitutive expressions of these genes under these conditions. However, only *narG1* expression was observed in the biofilm, which suggests differential expression mechanisms and roles for these two Nar systems (Auclair et al., 2012). Although open reading frame (ORF) encoding a nitrite reductase (NirS- or NirK-type) was absent in strain JAM1 genome, gene clusters encoding two nitric oxide reductases (Nor1 and Nor2) and one nitrous oxide reductase (Nos) were found. The gene clusters encoding for these five reductases (Nar1, Nar2, Nor1, Nor2 and Nos) and constituting an incomplete denitrification pathway are located in close proximity in a 67 kb chromosomic region (Villeneuve et al., 2013).

The importance of each Nar system in nitrate reduction and growth in *M. nitratireducenticrescens* JAM1 was assessed by generating *narG1* and *narG2* knockout mutants of strain JAM1. These mutants were tested for their abilities to grow and reduce nitrate under different conditions. Changes in the expression levels of the two *narGs* were then measured by reverse transcription-real-time PCR (RT-qPCR). We also measured the effects of such mutations on the expression levels of the nitrate transporter genes *narK*. Our results revealed that the two Nar systems contribute to growth and nitrate reduction but at different levels. Furthermore, the transcriptome of *M. nitratireducenticrescens* JAM1 that was grown under anaerobic conditions was derived to assess the expression level of the denitrification genes. Our results showed that the *nar1* gene cluster was eight times more expressed than the *nar2* gene cluster. Determination of the importance to these two nitrate reductases will help to decipher some of the mechanisms of denitrification in a marine environment.

## Materials and Methods

### Bacterial Strains and Growth Conditions

*Methylophaga nitratireducenticrescens* JAM1 (ATCC BAA-2433^T^, DSM 25689^T^) and the knockout mutants were cultured in the *Methylophaga* medium 1403 (Villeneuve et al., 2013). This medium contained 37 mM ammonium chloride. After autoclaving, methanol, solution T, Wolf’s mineral solution, vitamin B_12_ and nitrate were added under sterile conditions. Aerobic cultures were made in 200-ml Erlenmeyer flasks with constant shaking at 350 rpm. For the cultures under anaerobic conditions, 30 ml of non-sterile 1403 medium was dispensed into 70-ml serum bottles, which were then flushed for 15 min with N_2_ to remove oxygen from the headspace. The bottles were sealed with rubber stoppers and metal seals and autoclaved. Inoculum was made of fresh aerobic culture without nitrate to reach an optical density (OD_600nm_) of 0.025. Nitrate was added as sodium nitrate (NaNO_3_) at a final concentration of 40 mM. Biological replicates were conducted for all growth experiments and experiments were repeated once on a different day. The maximum specific growth rates were derived by non-linear regression with the Monod equation (Healey, 1980).

Bacterial growth was monitored by spectrophotometry (OD_600nm_). Samples were homogenized prior to measurements with a Potter-Elvehjem homogenizer to disperse flocs, which appeared in aerobic cultures of all strains. Nitrate and nitrite concentrations were determined by ion chromatography using the 850 Professional IC (Metrohm, Herisau, Switzerland) with a Metrosep^®^ A Supp 5 analytical column (250 mm × 4.0 mm). For the N_2_O reduction culture assays, strain JAM1 was cultured under anaerobic conditions with 40 mM NaNO_3_ and 100 μmol N_2_O injected in the headspace (3500 ppmv). Headspace accounted for approximately 40 ml, half of the total volume of the flask. N_2_O concentration in the headspace was determined by gaseous chromatography using an Agilent 7890B series GC Custom (SP1 option 7890-0504/0537) with a Haye Sep Q80/100 column. N_2_O was detected with an electron capture detector.

### Construction of the Knockout Mutants

A protocol modified from Thongdee et al. (2008) was used to construct the JAM1Δ*narG1* and the JAM1Δ*narG2* mutants. The upstream and downstream regions of *narG1* and *narG2* were amplified and subsequently fused together with two rounds of PCR (**Supplementary Image S1**). The JAM1Δ*narG1narG2* double mutant was obtained using the same protocol with JAM1Δ*narG1* as the host strain.

The first round of PCR was performed in a 50-μl reaction volume with the ThermoPol^®^ Buffer (New England Biolab), 10 μg bovine serum albumin (BSA), 200 μM deoxynucleotide triphosphate (dNTP), 100 pmol of each primer (**Supplementary Table S1**), 100 ng of strain JAM1 DNA and 1.25 U of Taq DNA polymerase. PCR was performed at 95°C for 30 s, followed by 30 cycles at 95°C for 30 s, 63.5°C or 64°C for 45 s for the Δ*narG1* or Δ*narG2* constructions, respectively, 68°C for 30 s (**Supplementary Table S1**), and a final extension period of 10 min at 68°C. The second round of PCR was performed with the same PCR reagents, 50 ng of each of the first round amplicons and 100 pmoles of the narG1-upF/narG1-dnR or narG2-upF/narG2-dnR primers (**Supplementary Table S1**). However, these primers were added only after the third PCR cycle to allow the two amplicons to join together. PCR was then performed as in the first PCR round. The expected DNA fragments were extracted by agarose gel electrophoresis and purified using Wizard^®^ SV Gel and PCR Clean-Up System (Promega). The fragments were ligated into the pEX18Gm plasmid vector (Hoang et al., 1998) via SacI and BamHI for the *narG1* knockout mutant and EcoRI and PstI for the *narG2* knockout mutant. The DNA constructions were then cloned in *Escherichia. coli* DH5α (Sambrook et al., 2001) and screened for gentamicin-resistant clones. Plasmid DNA was extracted from the representative clones, and the presence of the insert in the plasmid was verified by enzymatic digestion with SacI and BamHI or EcoRI and PstI.

The respective constructions were electroporated in strain JAM1 (or in the JAM1Δ*narG1* mutant for the double mutant) using the following protocol: strain JAM1 was cultured for 24 h to reach an OD_600nm_ of 0.6; 1 ml of the culture was centrifuged at 12,000 × *g* for 1 min; the cells were washed five times with 300 mM of a sucrose solution to remove all salts and then concentrated in 100 μl of sucrose solution; the electroporation was performed on ice with 100 μl of bacterial cells and 100 ng of plasmid DNA at 2500 V; after the electric shock, the cells were transferred in the 1403 medium and incubated at 30°C for 4 h and then centrifuged and plated on 1403 agar medium supplemented with gentamicin (50 μg/ml); the resistant colonies were transferred in 1403 medium supplemented with 50 μg/ml of gentamicin; and the simple recombinants were plated on 1403 agar medium supplemented with sucrose (10%) to isolate the double recombinants using the sucrose sensitivity gene *sacB* of pEX18Gm. The gene arrangements of the knockout mutants were confirmed by PCR. Attempts to do complementation assays in the mutants by electroporation or by conjugation with the MiniCTX1, MiniCTX2, and pUC18-miniTn7 integrative vectors were unsuccessful.

### RNA Extraction

To measure the transcriptional level of *narG1*, *narG2*, *narK1*, and *narK12f* by RT-qPCR, total RNA was extracted from strain JAM1 and the JAM1Δ*narG1* and JAM1Δ*narG2* mutants that were cultivated under the following conditions: (1) aerobic without nitrate, (2) aerobic with nitrate and (3) anaerobic with nitrate. Total RNA from strain JAM1 (anaerobic conditions with nitrate) was also used to investigate the possible co-transcription of *narK1narK2* and *nar[1]GHJI*, of *nar[2]GHJI* and *ppi*, and of *ppi* and *narK12f*. In all cases, samples (5–30 ml) were taken during the exponential growth phase (20–24 h of incubation). The bacteria were centrifuged and dispersed in the extraction solution (composed of 500 μl of phenol-Tris-HCl, pH 4.3 and 1.5 ml of 50 mM Tris-HCl, 100 mM EDTA, and 150 mM NaCl) and placed into 2-ml tubes containing 250 mg of 0.2 mm glass beads. The samples were then flash frozen in liquid nitrogen and stored at -80°C until extraction.

After thawing, RNA was extracted by bead beating twice for 20 s with a FastPrep-24^®^ (MP^TM^) set at 4.0. The tubes were then centrifuged at 16,000 × *g* for 15 min at 4°C, and 500 μl of the upper phase was extracted three times with a phenol/chloroform/isoamyl alcohol mix (25:24:1) and one time with chloroform/isoamyl alcohol (24:1). The RNA was then recovered by precipitation with 100 μl of 10 M ammonium acetate and 800 μl of 100% ethanol. After centrifugation at 16,000 × *g* for 15 min at 4°C, the supernatant was discarded, and the pellet was dried at room temperature. Finally, the RNA was dissolved in DEPC-treated water and stored at 4°C. The RNA extracts were treated twice with Turbo^TM^ DNase (Ambion) for 30 min to remove all contaminant DNA. The absence of DNA was verified by end-point PCR. Extractions were made separately from three independent samples.

### *Methylophaga nitratireducenticrescens* JAM1 Transcriptome

*Methylophaga nitratireducenticrescens* JAM1 transcriptome was derived from total RNA extracted of anaerobic cultures with methanol and 40 mM nitrate. RNA was sent to McGill University and Génome Quebec Innovation Centre for RNA sequencing by Illumina method. The Ribo-Zero^TM^ rRNA Removal Kits (Meta-Bacteria; Epicentre, Madison, WI, USA) were used to deplete total RNA of the ribosomal RNA. The RNA was then treated with the TruSeq Stranded mRNA Sample Prep Kit (Illumina Inc, San Diego, CA, USA). A total of 51,351,358, 49,962,176, and 52,021,554 reads were obtained for the three replicates after sequencing. All computations were made on the supercomputer Briarée from the Université de Montréal, managed by Calcul Québec and Compute Canada. Raw RNA-seq files have been deposited in the NCBI database (accession number SRP066381). Raw reads were filtered to remove low quality reads using FASTX toolkit^1^ by discarding any reads with more than 10% nucleotides with a PHRED score <20. Reads were then aligned with the reference genome *M. nitratireducenticrescens* JAM1 (GenBank accession number CP003390) using Bowtie (v 2.2.3) with default parameters. SAMtools (v 0.1.18) and BEDtools (v 2.20.1) were used for the generation of sam and bam files, respectively. The GC content of JAM1 genes was also calculated using BEDtools (v 2.20.1), prior to normalization. Normalization of the read count was done using the RPKM normalization function of the NOIseq package in R (Tarazona et al., 2011). To exclude features with low read counts, a low count filter was applied using a CPM method with a CPM value of 1 and a cutoff of 100 for the coefficient of variation.

Non-ribosomal RNA reads were associated with 3,095 of 3,096 annotated genes. The number of reads in corresponding genes between replicate cultures varied in the percentage of standard variation (%SD: SD*100/average number of reads) from 0 to 107%, with an average %SD of 12.2% and a median %SD of 9.7%. Only 48 annotated genes showed %SD above 40%. These results showed that, for the vast majority of genes, the expression pattern between replicates was consistent. Because methanol dehydrogenase is a key element of the metabolism in methylotrophs, the expression level of the reported genes in this study was normalized to the level of *mxaF* (10130 normalized reads), which was arbitrarily set at 100.

### RT-PCR

One step RT-PCRs were performed using QIAGEN^®^ OneStep RT-PCT Kits following the manufacturer’s protocol with 200 ng of total RNA extracted from strain JAM1 and 100 pmoles of the primers narK2-narG1-F/narK2-narG1-R, narI2-Ppi-F/narI2-Ppi-R, or Ppi-narK12f-F/Ppi-narK12f-R (**Supplementary Table S2**). Briefly, the reaction began with a reverse transcription step of 30 min at 50°C followed by an initial PCR activation step of 15 min at 95°C. Next, 30 cycles of 45 s at 94°C, 45 s at 60°C and 1 min at 72°C were performed with a final extension step of 10 min at 72°C. The amplicons were visualized by agarose gel electrophoresis and revealed by ethidium bromide staining. Negative controls were RT-PCR carried out with no template.

### RT-qPCR Assays

cDNAs were generated from the RNA using hexameric primers and the Reverse Transcription System from Promega (Madison, WI, USA) with 1 μg of RNA extract. Real-time quantitative PCR assays were performed with the PerfeCTa^®^ SYBR^®^ Green SuperMix ROX^TM^ (Quanta). The final volumes of all reactions were 20 μl, and the amplification mix was composed of 10 μl of PerfeCTa^®^ SYBR^®^ Green SuperMix, 0.4 μl of each primer (40 pmoles; **Supplementary Table S2**), 4.2 μl of RNA-free water and 5 μl of cDNA (25 ng). All reactions were performed in a Rotor-Gene 6000 real-time PCR machine (Corbett Life science). PCR began with an initial denaturation step of 10 min at 95°C followed by 40 cycles of 10 s at 95°C, 15 s at 60°C, and 20 s at 72°C. To confirm the purity of the amplified products, a melting curve was performed by increasing the temperature from 65 to 95°C in increments of 1°C per step with a pause of 5 s for each step. The reference genes used were *dnaG* (primase; locus tag: Q7A_342), *rpoD* (sigma factor, Q7A_343) and *rpoB* (RNA polymerase β subunit, Q7A_2329; **Supplementary Table S2**). These genes were retrieved from the strain JAM1 genome sequence (GenBank accession number CP003390). All genes for each sample were tested in a single run. The amplification efficiency was tested for each set of primer pairs by qPCR with a dilution of strain JAM1 DNA as the template. The amplification efficiencies for all primer pairs varied between 0.9 and 1.1.

### Relative Quantification

Relative quantifications were performed according to the ΔΔC_T_ method (Livak and Schmittgen, 2001). For each quantification calculation, only one reference gene was used as a control. The reference gene was chosen from among the three genes tested based on the lowest variability according to geNorm (Vandesompele et al., 2002), Normfinder (Andersen et al., 2004), BestKeeper (Pfaﬄ et al., 2004) and the comparative ΔΔC_T_ method (Silver et al., 2006). A final recommended comprehensive ranking was created from the geometrical means of the weights assigned by the four tests to allow for the selection of the appropriate control gene. The Prism software (version 5.00) was used for the statistical analyses, and significance was determined according to Student t-tests of the ΔC_T_s (α value = 0.05).

### Fnr and NarL DNA-Binding Motifs

The genomic sequence of *M. nitratireducenticrescens* JAM1 was analyzed in the upstream sequence of the *narX*, *narK1*, *narG2*, and *narK12f* for potential transcription factor binding sites (TFBSs) of Fnr/Anr and NarL regulators using the Virtual Footprint software of PRODORIC (Munch et al., 2005). Briefly, the software allowed the analysis of promoter sequences with position weight matrices (PWMs) of known TFBSs from the PRODORIC database^2^. A score was assigned to hits depending on the degrees of matches with the PWM. Potential regulator binding sequence scores were calculated with PWM. Threshold scores of 3.7, 5.0, and 7.0 were considered according to mean scores of PWMs for NarL (*E. coli*), NarL (*Pseudomonas aeruginosa*), and Fnr (*E. coli*), respectively.

## Results

### The Chromosomic Arrangement of the Denitrification Genes and their Expression Under Anaerobic Conditions

**FIGURE 1:**
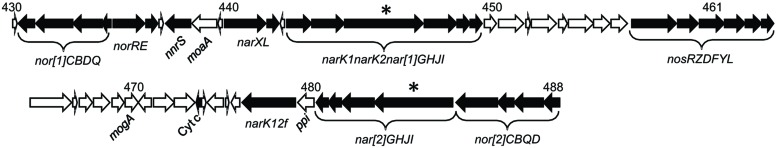
**Gene arrangement of the 67 kbp region containing all of the denitrification genes.** Sequences and annotations are from GenBank accession number CP003390.2. The numbers above the gene arrangement are the tag locus numbers (Q7A_0430 to Q7A_488). ^∗^The corresponding narG that were mutated to derive the knockout mutants. See **Supplementary Table S3** for the description of the 58 genes and the relative level of transcript reads.

The two *narGHJI* operons (*nar[1]GHJI* and *nar[2]GHJI*) that encode the Nar1 and Nar2 systems, respectively, are in close proximity and in opposite directions (**Figure 1**). Two ORFs encoding NarK transporters were found upstream of the *nar[1]GHJI* operon, and they corresponded, respectively, to the NarK1-type and NarK2-type transporters. NarK1 is a nitrate/proton symporter that uses the proton motive force to transport nitrate molecules into the cytoplasm, whereas NarK2 is a nitrate/nitrite antiporter that is able to uptake a nitrate molecule into the cytoplasm by releasing a nitrite molecule into the periplasm (Moir and Wood, 2001). The *narK* genes can be found upstream of the *narGHJI* operon in a large number of denitrifiers, and some species have a pair of *narKs* at this position, as in strain JAM1 (Schreiber et al., 2007). Another ORF encoding a fused NarK1-NarK2 transporter (here named *narK12f*; Goddard et al., 2008) is located downstream of the *nar[2]GHJI* operon. Between the *narK12f* and the *nar[2]GHJI* operons is an ORF encoding a parvulin-like peptidyl-prolyl isomerase (*ppi*). This type of protein is known to be involved in protein folding (Gothel and Marahiel, 1999). To highlight the potential co-transcription of *narK1narK2* and *nar[1]GHJI*, of *nar[2]GHJI* and *ppi*, and of *ppi* and *narK12f*, pairs of primers were designed to target the intergenic sequences of *narK2-narG1* (40 nt)*, narI2-ppi* (17 nt) and *ppi-narK12f* (18 nt) with RT-PCR. RT-PCR products were obtained for the intergenic regions of *narK2-narG1* but not for the intergenic region of *narI2-ppi* and of *ppi-narK12f* (**Supplementary Image S2**), which suggests co-transcription of *narK1narK2* with *nar[1]GHJI*.

Upstream of *narK1narK2nar[1]GHJI* are two ORFs that encode a two-component system similar to the nitrate/nitrite sensor system NarXL. The expressions of many genes are under control of this two-component system in *E. coli*, including the nitrate transporter *narK* and *narGHJI* (Li et al., 1994). NarL potential binding sites were found upstream of *narK1narK2nar[1]GHJI*, *nar[2]GHJI*, and *narXL* (**Table 1**).

**Table 1:** Sequences of potential NarL and ANR binding sites.

Upstream sequence	Site	Distance (nt)^∗^	Score	Sequence
*narKlnar K2nar[l]GHJI*	NarL	40	3.87	TACCGTT


	NarL	57	3.76	TACTCTA
	NarL	34	3.72	TACATCA
*nar[2] GHJI*	NarL	129	5.25	TACCGAT


	NarL	71	5.02	TGCTTCT
*narXL*	NarL	274	3.98	TACCGAT
	NarL	38	3.88	TACACCT
	NarL	134	3.79	TACTGTT
	NarL	232	5.35	TGCCGCT
	FNR/ANR	92	7.62	TTGATCAGGATCAA


*Upstream sequence were analyses with position weight matrices of known NarL binding sequence in *Pseudomonas aeruginosa* and *Escherichia coli* from the PRODORIC database. ^∗^Distance from the start codon.*

Strain JAM1 contains, in addition to the two *nar* gene clusters, two gene clusters predicted to encode two nitric oxide reductases (Nor) and one nitrous oxide reductase (Nos; **Figure 1**). Based on automated annotation, we reported previously that a small ORF (Q7A_474) encodes a truncated NirK (Villeneuve et al., 2013). Scrupulous analyses of this sequence revealed that it encodes a 137 amino acid protein with a cytochrome c binding site (CXXCH). Among the most related sequences are some NirK that contain this domain (Ellis et al., 2007). However, we did not find any related domains (e.g., copper binding sites) specific to NirK, which suggested that this ORF encodes cytochrome c. Sequence homology analyses in databases were negative for potential NirS/NirK in the strain JAM1 genome.

**FIGURE 2:**
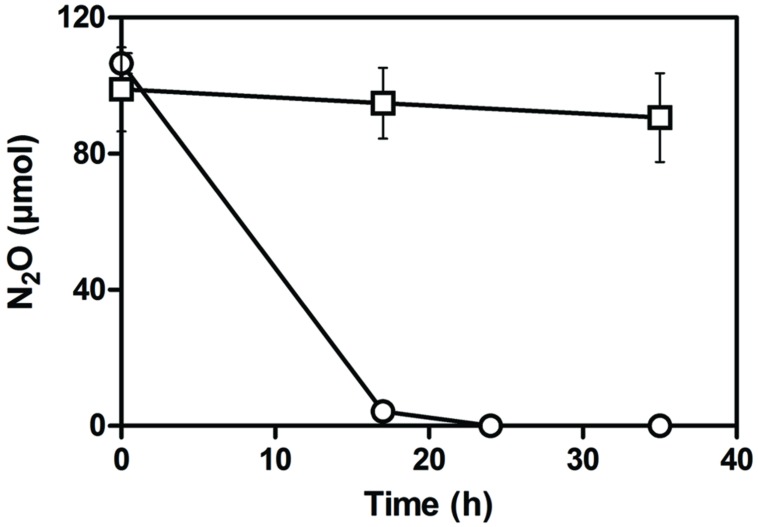
**N_2_O reduction by strain JAM1.** Strain JAM1 was cultured in triplicate under anaerobic conditions with nitrate (40 mM) and N_2_O (circle). A control with no biomass was also conducted (square).

Based on the transcriptome analysis, the gene expression level of the *nar1* gene cluster under anaerobic conditions is approximately eightfold higher than the *nar2* gene cluster and *narK12f* (**Table 2**). Despite the absence of a gene encoding nitrite reductase to generate NO, gene clusters encoding Nor1 and Nos were expressed at higher levels than the two *nar* gene clusters (**Table 2**). These results suggested that strain JAM1 can reduce NO and N_2_O. Indeed, strain JAM1 cultured under anaerobic conditions with nitrate and N_2_O can completely reduce N_2_O in 24 h (**Figure 2**).

**Table 2:** Relative level of transcriptomic reads of the denitrification genes.

Locus tag	Reads	Relative to *mxaF*^∗^	Gene name	Locus tag	Reads	Relative to *mxaF*^∗^	Gene name
**Nitrate reductase (Nar1), regulator and transporter**				**Nitrate reductase (Nar2) and transporter**			
	
Q7A_0441	589	5.8	*narX*	Q7A_0479	101	1.0	*narK12f*
Q7A_0442	511	5.0	*narL*	Q7A_0480	81	0.8	*ppi*
Q7A_0444	959	9.5	*narK*1	Q7A_0481	70	0.7	*narl*
Q7A_0445	591	5.8	*narK*2	Q7A 0482	117	1.2	*narJ*
Q7A_0446	871	8.6	*narG*	Q7A_0483	99	1.0	*narH*
Q7A_0447	697	6.9	*narH*	Q7A_0484	164	1.6	*narG*
Q7A_0448	948	9.4	*narJ*				
Q7A_0449	794	7.8	*narl*				

**Nitric oxide reductase (Norl)**				**Nitric oxide reductase (Nor2)**			
	
Q7A_0431	188	1.9	*norQ*	Q7A_0485	59	0.6	*norD*
Q7A_0432	268	2.7	*norD*	Q7A_0486	116	1.1	*norQ*
Q7A_0433	1432	14.1	*norB*	Q7A_0487	121	1.2	*norB*
Q7A_0434	1618	16.0	*norC*	Q7A_0488	195	1.9	*norC*
Q7A_0435	482	4.8	*norR*				
Q7A_0436	455	4.5	*norE*				

**Nitrous oxide reductase (Nos)**				**Genes related to denitrification**			
	
Q7A_0458	994	9.8	*nosR*	Q7A_0438	511	5.0	*nnrS*
Q7A_0459	1473	14.5	*nosZ*	Q7A_0066	830	8.2	*nnrS*
Q7A_0461	490	4.8	*nosD*	Q7A_1801	426	4.2	*nnrS*
Q7A_0462	280	2.8	*nosF*	Q7A_0067	2315	22.8	*nsrR*
Q7A_0463	262	2.6	*nosY*	Q7A_0409	138	1.4	*nsrR*
Q7A_0464	311	3.1	*nosL*	Q7A_0068	3481	34.4	*dnrN*
				07A_0307	401	4.0	*anr*


*Reads, Number of normalized reads. ^∗^Based on mxaF (10130 normalized reads) set at 100. NnrS, protein involved in response to NO; NsrR, Nitrite-sensitive transcriptional repressor; Anr, transcriptional regulator; DnrN or NorA, Nitric oxide-dependent regulator. Information was based on GenBank annotations (CP003390).*

Genes involved in the NO stress response, *nsrR* (two ORFs), *nnrS* (three ORFs) and *dnrN*, were found (**Table 2**), and all were expressed. NsrR is a transcriptional repressor from the Rrf2 family and it regulates the expression of multiple genes, such as *norB* in response to NO (Wang et al., 2008; Spiro, 2011). NnrS and DnrN are involved in protection against NO damage (Heurlier et al., 2008; Stern et al., 2013). These results suggested that strain JAM1 possesses different mechanisms, already expressed under anaerobic conditions, in response to potential NO damage.

### Effects of the Knockout Out Mutations of *narG1* and *narG2* on Growth and on Nitrate Reduction

**FIGURE 3:**
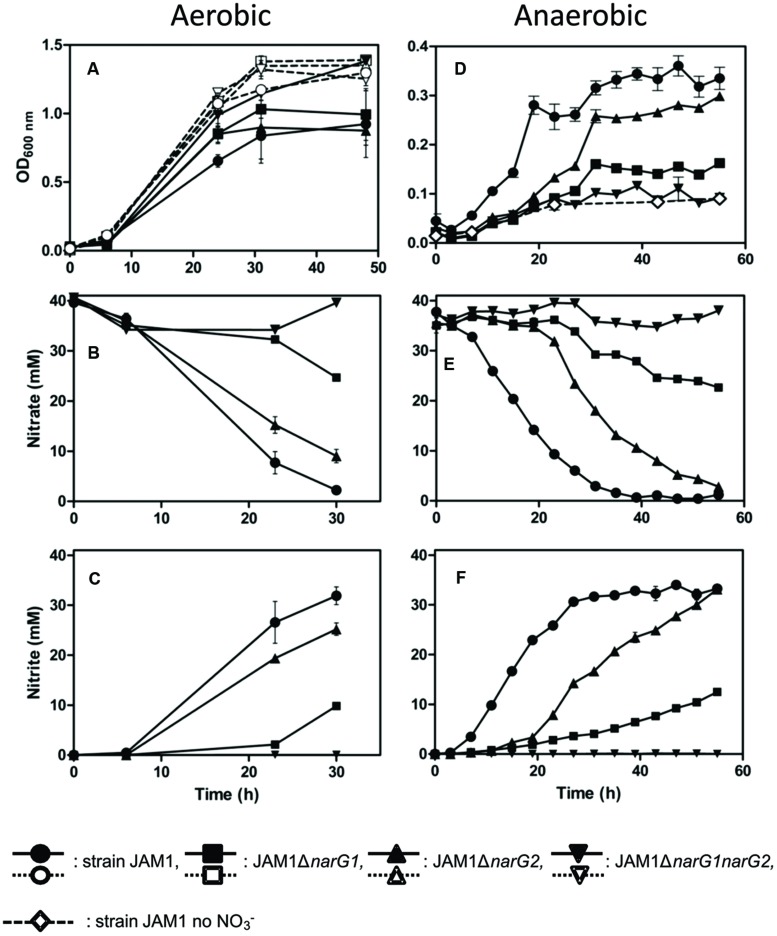
**Growth, nitrate reduction and nitrite production in cultures of strain JAM1 and the derivative *narG* mutants.** Strain JAM1 and the narG mutants were cultured under aerobic conditions without nitrate (**A**, dot lines), under aerobic conditions with nitrate (**A–C**, solid lines) or under anaerobic conditions with nitrate **(D–F)**, solid lines). Control with strain JAM1 and no nitrate was also conducted under anaerobic conditions (**D**, diamond with dash line). **(A)** and **(D)**: Growth. **(B)** and **(E)**: nitrate reduction. **(C)** and **(F)**: nitrite production.

The investigation of the importance of each Nar system in strain JAM1 was possible by establishing a knockout mutation protocol. The construction of single gene knockout mutants of the two *narGs* and a *narG1narG2* double mutant was achieved. The effects of each mutation on growth and nitrate reduction were investigated under three different conditions: aerobic without nitrate, aerobic with nitrate and anaerobic with nitrate (**Figure 3**).

Under aerobic conditions with nitrate (**Figures 3A–C**), strain JAM1 and the three mutants exhibited similar growth patterns (**Figure 3A**). Only the JAM1Δ*narG1narG2* double mutant cultures presented with a higher growth yield in the stationary phase. This growth yield was also reached by all strains when they were cultured under aerobic conditions without nitrate (**Figure 3A**). The deletion of *narG1* (JAM1Δ*narG1*) resulted in an 81% decrease in the nitrate specific reduction rate relative to strain JAM1, and the deletion of *narG2* (JAM1Δ*narG2*) resulted in a 36% reduction.

In contrast to the observations under aerobic conditions, strain JAM1 and the three mutants exhibited distinct growth patterns when cultured under anaerobic conditions (**Figure 3D**). The JAM1Δ*narG1narG2* double mutant exhibited a minimal level of growth which was also observed in the anaerobic nitrate-free culture controls (**Figure 3D**). Therefore, this growth was likely the result of residual O_2_ contained in the complement solution or introduced during sampling. The lack of the *narG2* gene led to a lower growth rate of the JAM1Δ*narG2* mutant, but the growth yield reached by this mutant was similar to that of strain JAM1. The JAM1Δ*narG1* mutant also exhibited a much lower growth rate, and did not reach the same maximum growth yield reached by the JAM1Δ*narG2* mutant and strain JAM1 after 55 h of culture. All cultures reached the stationary phase after 31 h of incubation.

The nitrate consumption of each strain appeared to be correlated to growth (**Figure 3E**). Strain JAM1 reduced all of the nitrate contained in the medium within only 40 h with no delay, although the precultures were grown under aerobic conditions without nitrate. The JAM1Δ*narG2* mutant only began to reduce nitrate after 20 h of incubation, which corresponded to the beginning of its exponential growth phase. Total nitrate reduction was achieved after 55 h of incubation. The JAM1Δ*narG1* mutant also exhibited a delay in nitrate reduction, which began after 23 h of incubation. The reduction of nitrate was still occurring during the late stationary phase. In all conditions, nitrite accumulated proportionally to nitrate reduction (**Figures 3C,F**). Finally, no reduction of nitrate or the production of nitrite was observed for the JAM1Δ*narG1narG2* double mutant under aerobic or anaerobic conditions, which demonstrated that no other dissimilatory nitrate reduction enzymes were active in strain JAM1, as predicted by the genome sequence.

Mass balance analysis of nitrate reduction and nitrite accumulation in strain JAM1 and the single mutants cultured under anaerobic conditions (**Figure 3**) showed 7% decrease in average nitrite concentration over 55 days (0.05 mM NO_3_-NO_2_ reduced h^-1^), with no apparent decrease in the abiotic controls. This slight decrease could be the result of the nitrate assimilation pathway present in strain JAM1 genome, and concurred with the low number of reads corresponding to this pathway present in the transcriptome (data not shown).

**FIGURE 4:**
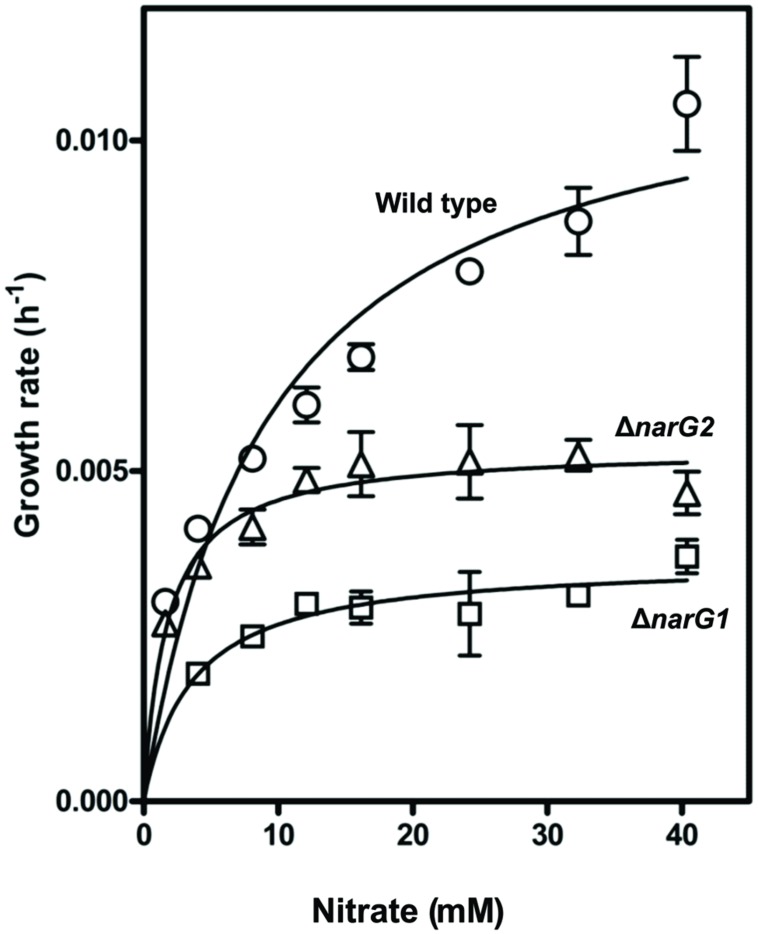
**Specific growth rates.** The specific growth rates of strain JAM1 and the *narG* mutants were derived from triplicate cultures of the same inoculum under anaerobic conditions.

Strain JAM1 and the JAM1Δ*narG1* and JAM1Δ*narG2* mutants were cultured under anaerobic conditions with different concentrations of nitrate to derive the maximum specific growth rates and the half-saturation constants of nitrate for growth (μ_max_ and *K*_s_; **Figure 4**). The μ_max_ of the JAM1Δ*narG1* and JAM1Δ*narG2* mutants were 3.2 and 2.2 times lower than the μ_max_ of strain JAM1, respectively (**Table 3**), which suggest additive effect of both Nar systems for growth. To assess the affinities of strain JAM1 and the mutants toward nitrate for growth, the μ_max_/*K*_s_ ratios were calculated (Healey, 1980). These ratios were in same order of magnitude ranging from 1.0 to 3.1.

**Table 3:** Kinetics of growth and nitrate reduction under denitrifying conditions.

	Strain JAM1	JAMI Δ*narGl*	JAMI Δ*narG2*
Specific growth rates			
μ_max_(h^-1^)	0.0116 (0.0008)	0.0036 (0.0003)	0.0053 (0.0002)
K_s_ (mM)	9.2 (1.9)	3.6 (1.2)	1.7 (0.4)
μ_max_/K_s_(mM^-1^ h^-1^)	1.3 (0.3)	1.0 (0.6)	3.1 (0.6)


*μ_max_, maximum specific growth rates. K_s_, half-saturation constants of nitrate for growth. Values between parentheses are standard deviation of triplicates.*

### Relative Expression Levels of *narG1, narG2, narK1*, and *narK12f*

Strain JAM1 and the JAM1Δ*narG1* and JAM1Δ*narG2* mutants were cultivated under aerobic conditions with or without nitrate and under anaerobic conditions, and the biomasses were collected at the end of the exponential phase when nitrate reduction activity was present in all strains. RT-qPCR assays were then performed to measure the relative transcript levels of the two *nar* gene clusters and *narK1* and *narK12f*. The transport of nitrate into the cytoplasm is a key parameter in nitrate reduction by membrane-bound nitrate reductases because the efficiency of nitrate reduction depends on their presence (Bonnefoy and Demoss, 1992; Ferguson, 1994; Moir and Wood, 2001; Sohaskey and Wayne, 2003; Clegg et al., 2006).

**FIGURE 5:**
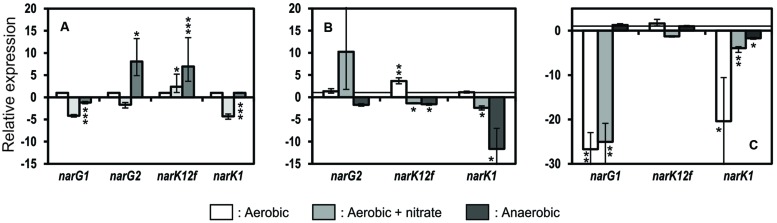
**Relative expressions of *narG1*, *narG2*, *narK12f*, and *narK1*.** Strain JAM1 **(A)**, JAM1Δ*narG1* **(B)** and JAM1Δ*narG2* **(C**) were cultured in triplicate of the same inoculum under aerobic conditions with or without nitrate and under anaerobic conditions with 40 mM nitrate. Biomass was collected during the exponential phase, and total RNA was extracted for RT-qPCR assays. **(A)** Changes in the levels of *narG1*, *narG2*, *narK12f*, and *narK1* transcripts in strain JAM1 cultures under aerobic with nitrate and anaerobic conditions were calculated relative to their expressions under aerobic conditions without nitrate (set to one, white column). **(B,C)** Changes in the levels of *narG1*, *narG2*, *narK12f*, and *narK1* transcripts in the JAM1Δ*narG1* and JAM1Δ*narG2* cultures under the three conditions were calculated relative to their expressions in the strain JAM1 cultures under the respective conditions. Error bar represents standard deviation of triplicate values. ^∗^0.05 < *P* < 0.01; ^∗∗^0.01 < *P* < 0.001; ^∗∗∗^*P* < <0.001.

The cultivation of strain JAM1 under aerobic conditions with nitrate led to a fourfold decrease in *narG1* and *narK1* expression (**Figure 5A**) relative to aerobic conditions without nitrate (reference culture set at one), whereas *narG2* and *narK12f* were expressed at a significantly greater level (eight and sevenfold increase, respectively) under anaerobic conditions. There were no significant differences in *narG2* expression levels between strain JAM1 and JAM1Δ*narG1* under any of the culture conditions (**Figure 5B**), whereas slight significant variations occurred in the expression of *narK12f*. The *narK1* expression in JAM1Δ*narG1* cultivated under anaerobic conditions with nitrate decreased by 11-fold relative to strain JAM1 cultivated under the same conditions. In JAM1Δ*narG2* cultivated under aerobic conditions with or without nitrate, the level of *narG1* and *narK1* transcripts was much lower (25- and 27-fold decrease for *narG1* and 20-and 4-fold decrease for *narK1*, respectively) than those in strain JAM1 cultivated under the same conditions (**Figure 5C**). Finally, no significant change was observed in the expression levels of *narK12f* between JAM1Δ*narG2* and strain JAM1 cultivated under any conditions (**Figure 5C**).

## Discussion

Genetic tools are essential for deciphering the different mechanisms of action of organisms. These tools are rarely readily available with new environmental bacterial species and they have to be derived from other systems or developed entirely. In this study, the investigation of the importance of the two Nar systems in strain JAM1 were possible by establishing a knockout mutation protocol. The strategy of building the *narG* gene knockout mutants was derived from that of Hoang et al. (1998), and this strategy has been successfully applied in *P. aeruginosa* (Déziel et al., 2004; Colvin et al., 2011; Jain and Kazmierczak, 2014). This study is the first report of *Methylophaga* gene knockout mutants. The targeting strategy developed in our study will certainly allow for the determination of the involvements of different *Methylophaga* genotypes in future studies. However, other genetic tools such as integrative vectors for gene complementation or autonomous plasmids for gene regulation studies have still to be developed.

The presence of the two Nar systems could allow strain JAM1 to reduce more nitrate in a defined time, which produces more energy for the cells and thus allows faster growth. The inactivation of each of the two Nar systems clearly showed that both systems contributed to the growth of strain JAM1 under anaerobic conditions, but at different levels. The differences between the two mutants in growth rate and nitrate reduction could be explained by differences in specific activity of the two nitrate reductases, by higher concentration of the Nar1 system in the membrane, or both. The calculated μ_max_/*K*_s_ ratios of the two mutants suggest that both Nar systems have similar specific activity toward nitrate for growth. On the other hand, the transcriptome of strain JAM1 cultivated under anaerobic conditions showed that the *nar1* gene cluster was eight times more expressed than the *nar2* gene cluster, suggesting higher concentration of the Nar1 system during these growth conditions. Interestingly, the Nar2 system seems to have another effect to the nitrate reduction dynamics as the lack of Nar2 action in JAM1Δ*narG2* induced the 20-h lag time prior to nitrate reduction that occurred in this mutant by the Nar1 system when cultured under anaerobic conditions.

Strain JAM1 demonstrated a large capacity in nitrate reduction, even under aerobic conditions. This would suggest a constitutive expression of genes involved in nitrate reduction or the lack of a functional oxygen regulation response. The single mutants and the wild strain cultured under aerobic conditions without nitrate reached higher growth yield than under aerobic conditions with nitrate (**Figure 3A**). These observations suggest that the nitrate and oxygen respiration systems in strain JAM1 compete with each other. This has also been suggested for *P. aeruginosa* (Chen et al., 2003). This would explain the highest growth yield reached by the JAM1Δ*narG1narG2* double mutant in aerobic cultures with or without nitrate (**Figure 3A**) as, without functional nitrate respiration, the oxygen respiration produced energy more efficiently. Competition between both respiration systems may explain the significant decrease of the *narG1* transcripts in strain JAM1 cultured under aerobic conditions with nitrate. Another suggestion would be that nitrite toxicity limits the growth of strain JAM1. Indeed, Auclair et al. (2010) showed a fourfold decrease in biomass when strain JAM1 was cultured aerobically in presence of 0.36 mM nitrite (no nitrate) at the beginning of the culture, and no growth occurred in the presence of 0.71 mM nitrite.

Oxygen and nitrate are known to regulate Nar expression in several bacteria. Fumarate and nitrate reductase regulation (FNR) protein family, an oxygen responsive transcription regulator, is known to be a major nitrate reductase regulator in several bacteria (Gunsalus and Park, 1994; Jordan et al., 1997; Philippot and Hojberg, 1999). Strain JAM1 encodes in its genome an ANR protein (**Table 2**), and one potential ANR binding site was found upstream of *narXL* (**Table 1**). Transcriptional regulation consistent with the presence of ANR/NarL was observed with the *nar2* gene cluster and *narK12f* under anaerobic conditions in strain JAM1, with an eightfold increase of their transcriptional level relative to the aerobic conditions (**Figure 5A**). Such a pattern was not observed with the *nar1* gene cluster, where *narG1* and *narK1* were not upregulated under anaerobic conditions compared to the aerobic conditions without nitrate. The high nitrate reduction capacity of strain JAM1 under both aerobic and anaerobic conditions suggests high level of the basal expression of nitrate reductases, in particular the Nar1 system (**Table 2**). However, in absence of the Nar2 system (JAM1Δ*narG2*), gene expression of *narG1* and *narK1* (both are co-transcribed) was deeply affected (*ca*. 25-fold decrease) by the presence of oxygen. In presence of oxygen and nitrate, gene expression of *narG1* and *narK1* of strain JAM1 was also down regulated but at a lower extend (fourfold decrease). These results suggest that the Nar2 system contributes in the regulation of the expression of the Nar1 system in the presence of oxygen in strain JAM1. How this regulation occurs (directly or indirectly) is unknown. There is still the possibility that this effect is an artifact created by the gene deletion. Regulation by *cis*- or *trans*-regulatory elements from the vector was excluded, because the three mutants were generated by double recombination and that no trace of the vector was found. Attempts to generate complement strains failed with three integrative vectors that were successfully used with other gamma-proteobacteria.

While it is not infrequent to find multiple nitrate reductases in a single bacterial species, the presence of multiple nitrate reductases of the same type has been reported only rarely in previous studies (Bonnefoy and Demoss, 1994; Chang et al., 1999; Potter et al., 1999; Chen et al., 2011; Hartsock and Shapleigh, 2011). For instance, *E. coli* expresses two different cytoplasmic membrane nitrate reductases, nitrate reductase A (NRA) and nitrate reductase Z (NRZ), which are encoded by two different operons, *narGHJI* and *narZYWV*, respectively (Bonnefoy and Demoss, 1994). The roles of these two Nar systems in the cell appear to be distinct because NRA reduction activity accounts for 98% of all of the nitrate reduction activity of *E. coli*. The expression of *narGHJI* is positively controlled by nitrate concentration through the NarXL two-component system and is strongly repressed by oxygen. Unlike what was observed in strain JAM1, growth of the *E. coli*Δ*narZ* mutant expressing only the NRA system is similar to that of the wild type strain in minimal medium in which nitrate is the sole final electron acceptor, while the *E. coli* Δ*narG* mutant expressing only the NRZ system is unable to grow under these conditions (Potter et al., 1999). In *Hyphomicrobium zavarzinii* that also encodes two Nar systems, the expression of only one *nar* gene cluster was deeply downregulated by oxygen (Martineau et al., 2015).

Although in pure culture, strain JAM1 accumulated nitrite, in the denitrifying biofilm, where it was isolated, nitrite is most likely processed by other denitrifying bacteria such as *H, nitrativorans* NL23, the second most represented bacteria in the biofilm (Labbé et al., 2007; Auclair et al., 2012; Martineau et al., 2013). Both bacteria are methylotroph, but assimilate differently the carbon. *Methylophaga* sp. process methanol via the RuMP pathway, whereas *Hyphomicrobium* sp. via the serine pathway. Other denitrifying bacteria were found in the biofilm but they represented less than 1% of total bacteria (data not shown). One model is that syntrophy was established in the biofilm between *M. nitratireducenticrescens* JAM1 and *H. nitrativorans* NL23 for denitrification. Also, because *Methylophaga* sp. are known to produce the osmo-protectant ectoine (Doronina et al., 2010), strain NL23 would have been protected against osmotic stress as it is intolerant to seawater in pure culture (Martineau et al., 2013, 2015). Genes involved in the production of ectoine were found among the most expressed in strain JAM1 pure culture (*ectABC*: 35–49 relative to *mxaF*).

## Conclusion

The transcriptional level of *nar1* was higher than *nar2* in strain JAM1 cultured under anaerobic conditions. The absence of the Nar1 system had a more negative effect on the growth rate and nitrate reduction rate than the absence of Nar2. These results suggested that the Nar1 system is the major nitrate reductase in strain JAM1. However, the Nar2 system appears to contribute in the regulation of the expression of the Nar1 system. Its absence induced a 20-h lag time in nitrate reduction. At the gene level, the *nar1* gene cluster is deeply downregulated by oxygen in the absence of the Nar1 system. In contrast, the absence of the Nar1 system did not affect the level of the *nar2* transcripts. New knowledge generated in this study about the importance of the two dissimilatory (Nar) nitrate reductases in *M. nitratireducenticrescens* JAM1 could allow for a better understanding of the denitrification pathway within the biofilm microbial population where strain JAM1 was isolated.

## Author Contributions

RV: Corresponding author; experimental design; result analysis, writing the article.

FM: Experimental design; performed the experimental; result analysis, writing the article.

CM: Experimental design; result analysis, writing the article.

## Conflict of Interest Statement

The authors declare that the research was conducted in the absence of any commercial or financial relationships that could be construed as a potential conflict of interest.

## Footnotes

1 http://hannonlab.cshl.edu/fastx_toolkit/

2 http://www.prodoric.de/

## References

[B1] Andersen C. L. Jensen J. L. Orntoft T. F. (2004). Normalization of real-time quantitative reverse transcription-PCR data: a model-based variance estimation approach to identify genes suited for normalization, applied to bladder and colon cancer data sets. *Cancer Res.* 64 5245–5250. 10.1158/0008-5472.CAN-04-0496 15289330

[B2] Auclair J. Lepine F. Parent S. Villemur R. (2010). Dissimilatory reduction of nitrate in seawater by a *Methylophaga* strain containing two highly divergent *narG* sequences. *Isme J.* 4 1302–1313. 10.1038/ismej.2010.47 20393572

[B3] Auclair J. Parent S. Villemur R. (2012). Functional diversity in the denitrifying biofilm of the methanol-fed marine denitrification system at the Montreal Biodome. *Microb. Ecol.* 63 726–735. 10.1007/s00248-011-9960-2 22006549

[B4] Boden R. (2012). Emended description of the genus Methylophaga Janvier et al. 1985. *Int. J. Syst. Evol. Microbiol.* 62 1644–1646. 10.1099/ijs.0.033639-0 21890722

[B5] Bonnefoy V. Demoss J. A. (1992). Identification of functional cis-acting sequences involved in regulation of *nark* gene-expression in *Escherichia coli*. *Mol. Microbiol.* 6 3595–3602. 10.1111/j.1365-2958.1992.tb01795.x 1474901

[B6] Bonnefoy V. Demoss J. A. (1994). Nitrate reductases in *Escherichia coli*. *Antonie Van Leeuwenhoek Int. J. Gen. Mol. Microbiol.* 66 47–56. 10.1007/Bf00871632 7747940

[B7] Chang L. Wei L. L. C. Audia J. P. Morton R. A. Schellhorn H. E. (1999). Expression of the *Escherichia coli* NRZ nitrate reductase is highly growth phase dependent and is controlled by RpoS, the alternative vegetative sigma factor. *Mol. Microbiol.* 34 756–766. 10.1046/j.1365-2958.1999.01637.x 10564515

[B8] Chen F. Xia Q. Ju L. K. (2003). Aerobic denitrification of *Pseudomonas aeruginosa* monitored by online NAD (P)H fluorescence. *Appl. Environ. Microbiol.* 69 6715–6722. 10.1128/AEM.69.11.6715-6722.2003 14602632 PMC262322

[B9] Chen Y. Wang F. Xu J. Mehmood M. A. Xiao X. (2011). Physiological and evolutionary studies of NAP systems in *Shewanella piezotolerans* WP3. *ISME J.* 5 843–855. 10.1038/ismej.2010.182 21124486 PMC3105760

[B10] Clegg S. J. Jia W. J. Cole J. A. (2006). Role of the *Escherichia coli* nitrate transport protein, NarU, in survival during severe nutrient starvation and slow growth. *Microbiol. SGM* 152 2091–2100. 10.1099/mic.0.28688-0 16804183

[B11] Colvin K. M. Gordon V. D. Murakami K. Borlee B. R. Wozniak D. J. Wong G. C. L. (2011). The Pel polysaccharide can serve a structural and protective role in the biofilm matrix of *Pseudomonas aeruginosa*. *PLoS Pathog.* 7:e1001264 10.1371/journal.ppat.1001264 PMC3029257 21298031

[B12] Déziel E. Lépine F. Milot S. He J. X. Mindrinos M. N. Tompkins R. G. (2004). Analysis of *Pseudomonas aeruginosa* 4-hydroxy-2-alkylquinolines (HAQs) reveals a role for 4-hydroxy-2-heptylquinoline in cell-to-cell communication. *Proc. Nat. Acad. Sci. U.S.A.* 101 1339–1344. 10.1073/pnas.0307694100 PMC337054 14739337

[B13] Doronina N. V. Ezhov V. A. Beschastnyi A. P. Iu A. (2010). Biosynthesis of the bioprotectant ectoin by aerobic methylotrophic bacteria from methanol. *Appl. Biochem. Microbiol.* 46 173–176. 10.1134/S0003683810020080 20391762

[B14] Ellis M. J. Grossmann J. G. Eady R. R. Hasnain S. S. (2007). Genomic analysis reveals widespread occurrence of new classes of copper nitrite reductases. *J. Biol. Inorg. Chem.* 12 1119–1127. 10.1007/s00775-007-0282-2 17712582

[B15] Ferguson S. J. (1994). Denitrification and its control. *Antonie Van Leeuwenhoek* 66 89–110. 10.1007/BF00871634 7747942

[B16] Goddard A. D. Moir J. W. B. Richardson D. J. Ferguson S. J. (2008). Interdependence of two NarK domains in a fused nitrate/nitrite transporter. *Mol. Microbiol.* 70 667–681. 10.1111/j.1365-2958.2008.06436.x 18823285

[B17] Gonzalez P. J. Correia C. Moura I. Brondino C. D. Moura J. J. (2006). Bacterial nitrate reductases: molecular and biological aspects of nitrate reduction. *J. Inorg. Biochem.* 100 1015–1023. 10.1016/j.jinorgbio.2005.11.024 16412515

[B18] Gothel S. F. Marahiel M. A. (1999). Peptidyl-prolyl cis-trans isomerases, a superfamily of ubiquitous folding catalysts. *Cell Mol. Life Sci.* 55 423–436. 10.1007/s000180050299 10228556 PMC11146858

[B19] Gunsalus R. P. Park S. J. (1994). Aerobic-anaerobic gene regulation in *Escherichia coli* - control by the ArcAB and Fnr regulons. *Res. Microbiol.* 145 437–450. 10.1016/0923-2508(94)90092-2 7855430

[B20] Hartsock A. Shapleigh J. P. (2011). Physiological roles for two periplasmic nitrate reductases in Rhodobacter sphaeroides 2.4.3 (ATCC 17025). *J. Bacteriol.* 193 6483–6489. 10.1128/JB.05324-11 21949073 PMC3232904

[B21] Healey F. P. (1980). Slope of the Monod equation as an indicator of advantage in nutrient competition. *Microb. Ecol.* 5 281–286. 10.1007/BF02020335 24232515

[B22] Heurlier K. Thomson M. J. Aziz N. Moir J. W. B. (2008). The nitric oxide (NO)-Sensing repressor NsrR of *Neisseria meningitidis* has a compact regulon of genes involved in NO synthesis and detoxification. *J. Bacteriol.* 190 2488–2495. 10.1128/JB.01869-07 18245279 PMC2293217

[B23] Hoang T. T. Karkhoff-Schweizer R. R. Kutchma A. J. Schweizer H. P. (1998). A broad-host-range Flp-FRT recombination system for site-specific excision of chromosomally-located DNA sequences: application for isolation of unmarked *Pseudomonas aeruginosa* mutants. *Gene* 212 77–86. 10.1016/S0378-1119(98)00130-9 9661666

[B24] Jain R. Kazmierczak B. I. (2014). A conservative amino acid mutation in the master regulator FleQ renders *Pseudomonas aeruginosa* aflagellate. *PLoS ONE* 9:e97439 10.1371/journal.pone.0097439 PMC4020848 24827992

[B25] Jordan P. A. Thomson A. J. Ralph E. T. Guest J. R. Green J. (1997). FNR is a direct oxygen sensor having a biphasic response curve. *FEBS Lett.* 416 349–352. 10.1016/S0014-5793(97)01219-1212 9373183

[B26] Labbé N. Laurin V. Juteau P. Parent S. Villemur R. (2007). Microbiological community structure of the biofilm of a methanol-fed, marine denitrification system, and identification of the methanol-utilizing microorganisms. *Microb. Ecol.* 53 621–630. 10.1007/s00248-006-9168-z 17394042

[B27] Li J. Kustu S. Stewart V. (1994). In vitro interaction of nitrate-responsive regulatory protein NarL with DNA target sequences in the fdnG, narG, *narK* and frdA operon control regions of *Escherichia coli* K-12. *J. Mol. Biol.* 241 150–165. 10.1006/jmbi.1994.1485 8057356

[B28] Livak K. J. Schmittgen T. D. (2001). Analysis of relative gene expression data using real-time quantitative PCR and the 2-DDCt method. *Methods* 25 402–408. 10.1006/meth.2001.1262 11846609

[B29] Martineau C. Mauffrey F. Villemur R. (2015). Comparative analysis of denitrifying activity in *Hyphomicrobium nitrativorans*, *Hyphomicrobium denitrificans* and *Hyphomicrobium zavarzinii*. *Appl. Environ. Microbiol.* 81 5003–5014. 10.1128/AEM.00848-15 25979892 PMC4495217

[B30] Martineau C. Villeneuve C. Mauffrey F. Villemur R. (2013). *Hyphomicrobium nitrativorans* sp. nov., isolated from the biofilm of a methanol-fed denitrification system treating seawater at the Montreal Biodome. *Int. J. Syst. Evol. Microbiol.* 63 3777–3781. 10.1099/ijs.0.048124-0 23667138

[B31] Moir J. W. B. Wood N. J. (2001). Nitrate and nitrite transport in bacteria. *Cell Mol. Life Sci.* 58 215–224. 10.1007/PL00000849 11289303 PMC11146482

[B32] Munch R. Hiller K. Grote A. Scheer M. Klein J. Schobert M. (2005). Virtual footprint and PRODORIC: an integrative framework for regulon prediction in prokaryotes. *Bioinformatics* 21 4187–4189. 10.1093/bioinformatics/bti635 16109747

[B33] Pfaﬄ M. W. Tichopad A. Prgomet C. Neuvians T. P. (2004). Determination of stable housekeeping genes, differentially regulated target genes and sample integrity: BestKeeper - Excel-based tool using pair-wise correlations. *Biotechnol. Lett.* 26 509–515. 10.1023/B:Bile.0000019559.84305.47 15127793

[B34] Philippot L. Hojberg O. (1999). Dissimilatory nitrate reductases in bacteria. *Biochim. Biophys. Acta* 1446 1–23. 10.1016/S0167-4781(99)00072-X 10395915

[B35] Potter L. C. Millington P. Griffiths L. Thomas G. H. Cole J. A. (1999). Competition between *Escherichia coli* strains expressing either a periplasmic or a membrane-bound nitrate reductase: does Nap confer a selective advantage during nitrate-limited growth? *Biochem. J.* 344 77–84. 10.1042/0264-60213440077 10548536 PMC1220616

[B36] Sambrook J. MacCallum P. Russell D. (2001). *Molecular Cloning: A Laboratory Manual*, 3rd Edn New York, NY: Cold Spring Harbor Laboratory.

[B37] Schreiber K. Krieger R. Benkert B. Eschbach M. Arai H. Schobert M. (2007). The anaerobic regulatory network required for *Pseudomonas aeruginosa* nitrate respiration. *J. Bacteriol.* 189 4310–4314. 10.1128/JB.00240-07 17400734 PMC1913380

[B38] Silver N. Best S. Jiang J. Thein S. L. (2006). Selection of housekeeping genes for gene expression studies in human reticulocytes using real-time PCR. *BMC Mol. Biol.* 7:33 10.1186/1471-2199-7-33 PMC1609175 17026756

[B39] Sohaskey C. D. Wayne L. G. (2003). Role of *narK2X* and *narGHJI* in hypoxic upregulation of nitrate reduction by *Mycobacterium tuberculosis*. *J. Bacteriol.* 185 7247–7256. 10.1128/Jb.185.24.7247-7256.2003 14645286 PMC296237

[B40] Spiro S. (2011). “Nitric oxide metabolism: physiology and regulatory mechanisms,” in *Nitrogen Cycling in Bacteria*, ed. Moir J. W. B. (Norfolk: Caister Academic Press), 179–196.

[B41] Stern A. M. Liu B. B. Bakken L. R. Shapleigh J. P. Zhu J. (2013). A novel protein protects bacterial iron-dependent metabolism from nitric oxide. *J. Bacteriol.* 195 4702–4708. 10.1128/JB.00836-13 23935055 PMC3807435

[B42] Tarazona S. Garcia-Alcalde F. Dopazo J. Ferrer A. Conesa A. (2011). Differential expression in RNA-seq: a matter of depth. *Genome Res.* 21 2213–2223. 10.1101/gr.124321.111 21903743 PMC3227109

[B43] Thongdee M. Gallagher L. A. Schell M. Dharakul T. Songsivilai S. Manoil C. (2008). Targeted mutagenesis of *Burkholderia thailandensis* and *Burkholdetia pseudomallei* through natural transformation of PCR fragments. *Appl. Environ. Microbiol.* 74 2985–2989. 10.1128/AEM.00030-08 18310423 PMC2394929

[B44] Vandesompele J. De Preter K. Pattyn F. Poppe B. Van Roy N. De Paepe A. (2002). Accurate normalization of real-time quantitative RT-PCR data by geometric averaging of multiple internal control genes. *Genome Biol.* 3 1–11. 10.1186/gb-2002-3-7-research0034 PMC126239 12184808

[B45] Villeneuve C. Martineau C. Mauffrey F. Villemur R. (2013). *Methylophaga nitratireducenticrescens* sp. nov. and *Methylophaga frappieri* sp. nov., isolated from the biofilm of the methanol-fed denitrification system treating the seawater at the Montreal Biodome. *Int. J. Syst. Evol. Microbiol.* 63 2216–2222. 10.1099/ijs.0.044545-0 23148104

[B46] Wang W. Richardson A. R. Martens-Habbena W. Stahl D. A. Fang F. C. Hansen E. J. (2008). Identification of a repressor of a truncated denitrification pathway in *Moraxella catarrhalis*. *J. Bacteriol.* 190 7762–7772. 10.1128/JB.01032-08 18820017 PMC2583601

