# Supplementary material for: Importance of the Two Dissimilatory (Nar) Nitrate Reductases in the Growth and Nitrate Reduction of the Methylotrophic Marine Bacterium Methylophaga nitratireducenticrescens JAM1
Source: Front Microbiol. 2015 Dec 24;6:1475. doi: 10.3389/fmicb.2015.01475 (PMC4689864; doi:10.3389/fmicb.2015.01475)
Supplement: Supplementary file 1 [file Image_1.PDF]

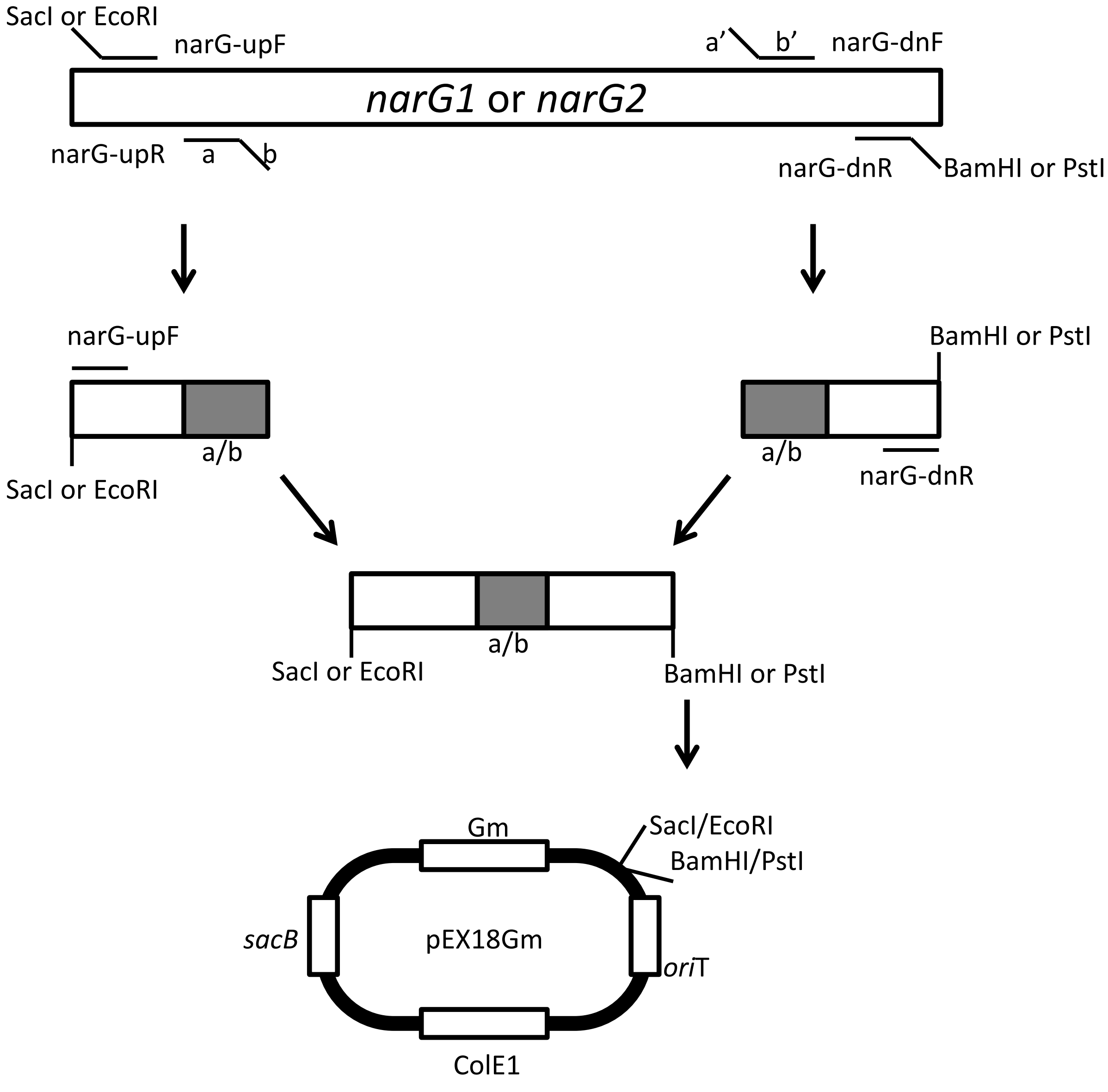

Figure S1. Schematic illustration of the generation of the mutant fragments and their cloning in the plasmid vector pEX18Gm.

In the first PCR round, the upstream and downstream regions of *narG1* and *nar*G2 were amplified by PCR with the respective upF/upR and dnF/dnR primers (Table S1). In the second PCR round, the upstream and downstream fragments were mixed and amplified with PCR with the upF/dnR primers. The resultant DNA fragment was ligated at the SacI sites of pEX18Gm and cloned in *E. coli* DH5α. The restriction sites used in the construction of the *narG1* mutants were SacI and BamHI, and EcoRI and PstI were used in the construction of the *narG2* mutants. Gm: gentamicin resistance gene. «a-a’» and «b-b’» are respective complementary sequences (see Table S1).
