# Supplementary material for: Importance of the Two Dissimilatory (Nar) Nitrate Reductases in the Growth and Nitrate Reduction of the Methylotrophic Marine Bacterium Methylophaga nitratireducenticrescens JAM1
Source: Front Microbiol. 2015 Dec 24;6:1475. doi: 10.3389/fmicb.2015.01475 (PMC4689864; doi:10.3389/fmicb.2015.01475)
Supplement: Supplementary file 2 [file Image_2.PDF]

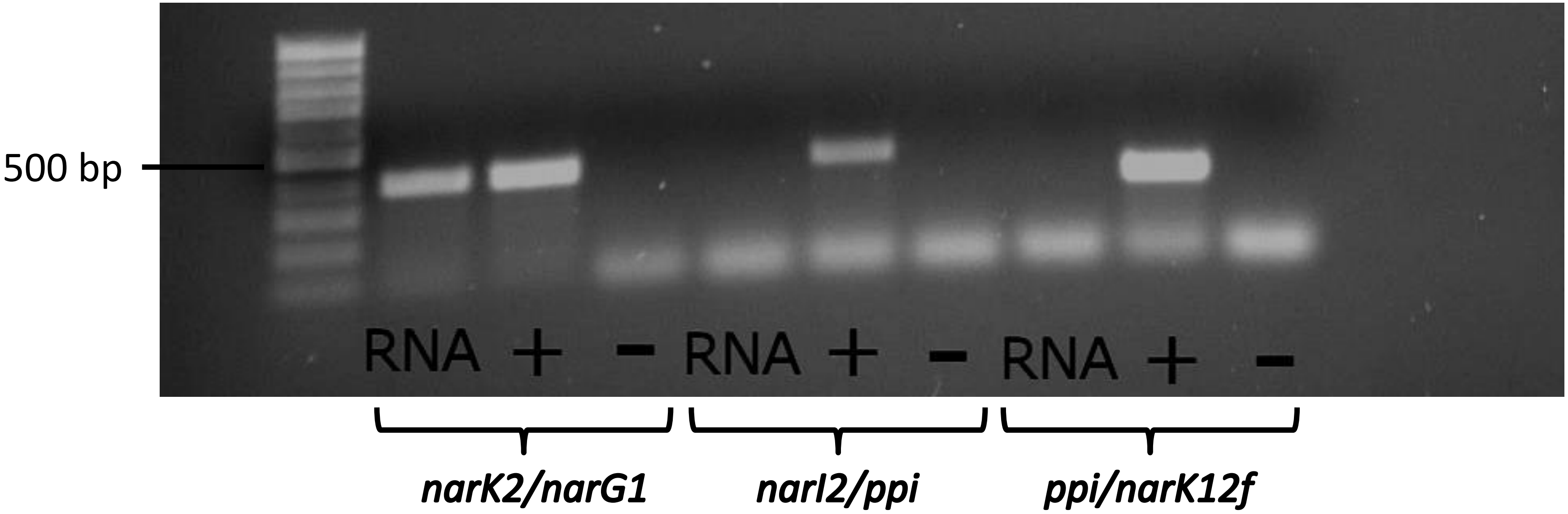

Figure S2. Determination of the transcription in the intergenic region between *narK2* and *narG1*, *narI2* and *ppi* and *ppi* and *narK12f*

RT-PCRs were performed with total RNA and primers (Table S2) that flanked the intergenic region *narK2* and *narG1*, *narI2* and *ppi* and *ppi* and *narK12f*. Amplicons were assessed with 1.5% agarose gel electrophoresis. RNA: RT-PCR with total RNA. +: RT-PCR positive control with JAM1 genomic DNA. (-): RT-PCR negative control with RNA-free water.
