# Supplementary material for: Importance of the Two Dissimilatory (Nar) Nitrate Reductases in the Growth and Nitrate Reduction of the Methylotrophic Marine Bacterium Methylophaga nitratireducenticrescens JAM1
Source: Front Microbiol. 2015 Dec 24;6:1475. doi: 10.3389/fmicb.2015.01475 (PMC4689864; doi:10.3389/fmicb.2015.01475)
Supplement: Supplementary file 3 [file Table_1.DOCX]

**Table S1. Primers used for the construction of the knockout mutants.**

Primers Sequence 5’-3’ Hybridization Description

temperature

Construction of the *narG1* mutant

narG1-upF^1^ ATCCATGAGCTCTTTTGTCGGAGCGTTGATT 63.5°C *narG1* upstream gene fragment

SacI

narG1-upR^2^ ttagcgtttaccgacccctattggcaagcggtctagaaaatgactcat *narG1* upstream gene fragment

a b

narG1-dnF^2^ atgagtcattttctagaccgcttgCCAATAGGGGTCGGTAAACGCTAA *narG1* downstream gene fragment

b' a'

narG1-dnR^1^ ATCCATGGATCCCGTTATCTGTGAGATAGGTCGTGC *narG1* downstream gene fragment

BamHI

Construction of the *narG2* mutant

narG2-upF^1^ ATCCACGAATTCGCCGAGTATTTCCTCCCAAT 64°C *narG2* upstream gene fragment

EcoRI

narG2-upR^2^ atgagaaccgtgaatgggaaaaatcggcatggtgcttaac *narG2* upstream gene fragment

a b

narG2-dnF^2^ gttaagcaccatgccgatttTTCCCATTCACGGTTCTCAT *narG2* downstream gene fragment

b' a'

narG2-dnR^1^ ATCCATCTGCAGGGCATTTTCGCAGACATTTT *narG2* downstream gene fragment

PstI

^1^ Underlined nucleotides are the restriction sites used for cloning in the pEX18Gm plasmid vector.

^2^ The a and a' sequences (uppercase), and the b and b' sequences (lowercase) are complementary sequences.
