# Supplementary material for: Importance of the Two Dissimilatory (Nar) Nitrate Reductases in the Growth and Nitrate Reduction of the Methylotrophic Marine Bacterium Methylophaga nitratireducenticrescens JAM1
Source: Front Microbiol. 2015 Dec 24;6:1475. doi: 10.3389/fmicb.2015.01475 (PMC4689864; doi:10.3389/fmicb.2015.01475)
Supplement: Supplementary file 4 [file Table_2.DOCX]

**Table S2. Primers used in RT-PCR and RT-qPCR.**

Primers Sequence 5’-3’ Description

narG1 (276F) GCTTTCCGCATTTCAACCTG RT-qPCR of *narG1*

narG1 (433R) ATTACCGCCACCCAGTTT

narG2 (597F) TTACGCTGCAGGATCACGTT RT-qPCR of *narG2*

narG2 (723R) TGACTCGGGTACATCGGTCT

narK12f (257F) TTCTGATCTGCCCGAACTCT RT-qPCR of *narK12f*

narK12f (362R) GCGCCTAGCAATGCTTTTAC

narK1 (490F) GTGCCAAAGGTTTACGCAAT RT-qPCR of *narK1*

narK1 (584R) ATTGCTTTGGCGACCTTATG

rpob (3861F) TGAGATGGAGGTTTGGGCAC RT-qPCR of *rpoB*

rpob (4006R) GCATACCTGCATCCATCCGA

rpoD (10F) CAGCAATCACGCGTTAAAGA RT-qPCR of *rpoD*

rpoD(153R) ACCCAGGTCGCTGAACATAC

dnaG (774F) CATCCTGATCGTGGAAGGTT RT-qPCR of *dnaG*

dnaG (894R) GCTGCGAATCAACTGACGTA

narK2-narG1-F CCCGGCAATCATTAATCAAC RT-PCR of polycistron

narK2-narG1-R GAATGACACCAAGCCGTTTT *narK2-narG1*

narI2-Ppi-F TTCTCGGTCAGCTTTTGCTT RT-PCR of polycistron

narI2-Ppi-R AAATGCAGCATCATGAGACG *narI2-ppi*

Ppi-narK12f-F TGAGGCCGAGATAGAGGGTA RT-PCR of polycistron

Ppi-narK12f-R CCGTCTGCCACCAAGATAAT *ppi-narK12f*

Hybridization temperature in PCR was 60°C for all pairs of primers
